# Genotype–environment interaction of crocin in *Gardenia jasminoides* by AMMI and GGE biplot analysis

**DOI:** 10.1002/fsn3.3003

**Published:** 2022-07-27

**Authors:** Qian Liu, Lili Huang, Chao Fu, Ting Zhang, Wei Ding, Chunxia Yang

**Affiliations:** ^1^ Jiangxi Academy of Forestry Nanchang Jiangxi P.R. China

**Keywords:** AMMI, crocin, *Gardenia jasminoides*, genotype–environment interaction, GGE biplot

## Abstract

To investigate the effect of genotype–environment interaction (GEI) on *Gardenia jasminoides* crocin contents, this study combined the additive main effects and multiplicative interactions (AMMI) model and genotype × environment interaction (GGE) biplot method to investigate the adaptation and stability of 11 *Gardenia jasminoides* genotypes at three experimental sites in Jiangxi Province with replications. The AMMI analysis showed that genotype, environment, and the GEI had extremely significant effects on *Gardenia jasminoides* crocin contents (*p* < .001). The GEI was the main factor causing the content variation, followed by genotype. The AMMI model and biplot analysis showed that the HC20 genotype had high and stable crocin contents. The GGE biplot analysis showed that Fengcheng and Gongqingcheng were optimal test sites for the selection of *Gardenia jasminoides* genotypes based on their crocin contents; additionally, the HC20 genotype was a suitable dominant genotype to promote cultivation in the test areas, and the GY8 genotype could be widely planted in the Gongqingcheng area. Therefore, the AMMI and GGE biplot genotype evaluation results were basically consistent. Comprehensive and effective evaluation of genotype and regional test sites can provide a theoretical basis for the breeding and development of *Gardenia jasminoides* clones with high and stable crocin contents and the selection of test sites.

## Graphical abstract

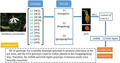

## 1 INTRODUCTION

*Gardenia jasminoides* is a traditional medicinal plant. The secondary metabolite crocin in its fruit is an apo‐carotenoid compound capable of antioxidative, anti‐inflammatory, antitumor, and blood lipid lowering effects (Asdaq & Inamdar, 2010; Hashemzaei et al., 2020; Lee et al., 2005; Lu et al., 2015) and is a nontoxic and stable natural food coloring (Hong et al., 2015). *Crocus sativus* stigma is the main raw material for the extraction of crocin, but the yield of *C. sativus* stigma is very low, so the price of crocin is very expensive. *G. jasminoides* has a wide distribution, large planting area, and high fruit yield, and the content of crocin in its fruit is very high. Therefore, it is particularly important to breed and popularize *G. jasminoides* clones with a high and stable yield of crocin.

Plant secondary metabolites are not only a series of useful natural products but also play a crucial role in plant environmental adaptability and defense (Yang et al., 2018). The synthesis and accumulation of plant secondary metabolites are determined by a combination of internal genetic and external environmental factors (Thakur et al., 2019). In recent years, research on crocin has mainly focused on its antioxidant capacity, extraction process optimization, suitable environmental conditions, and biosynthesis pathways (Cardone et al., 2019; Chen et al., 2008; Ji et al., 2017; Yang et al., 2009), but there are few reports on the effects of genotype–environment interaction (GEI) on the stability and adaptability of crocin contents, especially in *G. jasminoides*.

Genotype–environment interaction is the different responses and departures from the genetic master effect exhibited by genotypes under different environmental conditions. The economic yield and secondary metabolite content of most medicinal plants are susceptible to GEI (Beleggia et al., 2013; Lal et al., 2021). Two commonly used and effective multivariate models for analyzing GEI are additive main effects and multiplicative interactions (AMMI) models as well as genotype main effect and genotype × environment interaction (GGE) analysis (Shahriari et al., 2018). These models are used to evaluate the high yield and stability of plants and can be used to find ideal test sites. The AMMI model combines analysis of variance (ANOVA) and principal component analysis (PCA), which can efficiently analyze the most important factors causing variation. The AMMI model has been proven to be a powerful tool for describing GEI patterns (Hagos & Abay, 2013). The GGE biplot method selects representative genotypes and environments by analyzing the main effects of genotype and GEI. Due to the multivariable response of genotypes to multiple environments, multiple models should be used to improve the scientificity and accuracy of regional test data analysis (Gauch et al., 2008; Lal et al., 2021).

Therefore, this study combined the AMMI model and GGE biplot method to explore the GEI effect on the crocin content in *G. jasminoides* and screened ideal test sites and *G. jasminoides* genotypes.

## 2 MATERIALS AND METHODS

### 2.1 Experimental design

Eleven superior clones of *G. jasminoides*, mainly cultivated in Jiangxi Province, were selected for determination trials, which were carried out at three sites in Jiangxi, namely, Zhangshu (115.23°E, 28.05°N), Fengcheng (115.76°E, 27.99°N), and Gongqingcheng (115.78°E, 29.19°N). A randomized complete block design was adopted with 3 replicates for each clone and 10 plants for each repetition. Fruits of 11 clones were picked from October 24 to October 26, and they should be steamed in an electric rice cooker for 8 min to dry according to the requirements of the “Chinese Pharmacopoeia,” and then removed for further drying.

The meteorological data for the three sites were obtained from the meteorological stations of the sampling sites (Table 1). The annual precipitation at Gongqingcheng was much lower than those at Zhangshu and Fengcheng, and the sunshine duration was much longer than those at the other two points.

**TABLE 1:** Environmental overview of test sites

Climate index	E1 (Zhangshu)	E2 (Fengcheng)	E3 (Gongqingcheng)
Annual precipitation (mm)	1551.30	1498.10	1303.70
Annual mean temperature (°C)	18.68	19.20	18.47
Minimum monthly average temperature (°C)	5.50	5.52	4.70
Maximum monthly average temperature (°C)	29.90	30.28	30.40
Annual sunshine duration (h)	1624.90	1583.50	1747.80
Relative humidity (%)	78.84	75.85	78.50

The HC genotype was from Fuangcheng town, Fengcheng city, Jiangxi Province; the YF genotype was from Lugang town, Yongfeng County, Jiangxi Province; and the GY genotype was from Gaoyu town, Anji County, Zhejiang Province. All clones were propagated from cuttings in 2012 and were transplanted into 3 regional test sites in March 2013. From 2013 to 2017, conventional fertilization management was carried out in accordance with Jiangxi provincial standard technical regulations for standardized planting of *G. jasminoides* (DB36/T 694‐2012). After 2018, fertilization management was not carried out in the three areas for four consecutive years.

### 2.2 Determination of crocin contents

The crocin I content in *G. jasminoides* fruit was determined by high‐performance liquid chromatography (HPLC). Preparation of the reference solution: A total of 2.8 mg of crocin I was weighed and put into a 100 ml volumetric flask. Preparation of the test solution: Approximately 0.35 g of *G. jasminoides* medicinal powder was accurately weighed and placed into 100 ml volumetric flasks with 50% ethanol added to a constant volume. The flasks were ultrasonically extracted for 30 min, cooled through a 0.45 μm microporous filter membrane, and filtered. Chromatographic conditions: Shimadzu LC‐20AT HPLC, Waters C18 column (250 × 4.6 mm, 5 μm), mobile phase was acetonitrile (A):1% acetic acid water (B) gradient elution, the flow rate was 1.0 ml/min, the detection wavelength was 440 nm, the injection volume was 20 μl, and the column temperature was 30°C.

### 2.3 Statistical analysis

The agricolae package in R software was used to analyze the GEI effect on the crocin content in *G. jasminoides* by the AMMI model.

$$Y_{\mathrm{ij}}=\mu +G_{i}+E_{j}+\sum_{k}\lambda_{k}b_{\mathrm{ik}}z_{\mathrm{jk}}+\varepsilon_{\mathrm{ij}}$$

where *Y*_*ij*_ is the yield of genotype *i* in environment *j*, *μ* is the overall mean, *G*_i_ denotes genotype deviation, *E*_j_ indicates environment deviation, *λ*_k_ is the singular value for component *k*, *b*_*ik*_ is the Variety score, *z*_*jk*_ is the environmental score, and *Ɛ*_*ij*_ is the test residual (Rad et al., 2013).

The GGE biplot method was used to analyze the high yield and stability with Genstat 21 software. The GGE biplot analysis followed the formula:

$$Y_{\mathrm{ij}}-\mu -E_{j}=\lambda_{1}\varepsilon_{i1}\eta_{1j}+\lambda_{2}\varepsilon_{i2}\eta_{2j}+e_{\mathrm{ij}}$$

where *Y*_*ij*_ is the corresponding variable of the ith genotype in the jth environment, *μ* is the total mean, *E*_*j*_ is the main effect of the jth environment, *λ*_1_ and *λ*_2_ are singular values of principal components PC1 and PC2, and *ε*_*i*1_ and *ε*_*i*2_ are eigenvectors in the jth environment for PC1 and PC2 of the ith genotype in the jth environment (Mitroviã et al., 2012).

## 3 RESULTS

### 3.1 Variation analysis of crocin contents

The results (Table 2) showed that the total variation in crocin content in different environments was as follows, Gongqingcheng (E3) > Fengcheng (E2) > Zhangshu (E1), and the coefficients of variation were 32.07%, 28.52%, and 21.28%, respectively. The order of the variation coefficient of crocin content for each genotype was as follows: G9 > G11 > G3 > G1 > G5 > G10 > G2 > G7 > G6 > G8 > G4. In this study, the total coefficient of variation for the YF6 (G9) genotype was as high as 50.22%, and its content in Gongqingcheng (E3) was 3 times higher than that in Zhangshu (E1), indicating that this genotype may be greatly affected by the environment. The average crocin content of the HC20 (G4) genotype was as high as 11.19 mg/g, which was much higher than those of the other genotypes, and its coefficient was the lowest at only 7%. Moreover, the crocin content of the HC20 (G4) genotype was higher in all three environments, suggesting that this genotype was less affected by the environment.

**TABLE 2:** Variation analysis of crocin contents

Code	Content of crocin		
	Mean	SD	CV (%)
G1 (HC2)	7.68	2.34	30.50
G2 (HC3)	8.67	1.63	18.84
G3 (HC18)	7.58	2.58	34.10
G4 (HC20)	11.19	0.78	7.00
G5 (YF2)	5.97	1.75	29.32
G6 (YF3)	6.61	0.90	13.62
G7 (YF4)	6.86	1.15	16.72
G8 (YF5)	7.93	1.05	13.25
G9 (YF6)	8.90	4.47	50.22
G10 (YF7)	8.48	1.74	20.55
G11 (GY8)	9.45	4.22	44.59
E1 (Zhangshu)	7.24	1.54	21.28
E2 (Fengcheng)	7.78	2.22	28.52
E3 (Gongqingcheng)	9.34	3.00	32.07

Abbreviations: CV, Coefficient of variation; SD, standard deviation.

### 3.2 AMMI model construction and analysis

The AMMI model analysis of the GEI at each test site (Table 3) showed that the crocin content in *G. jasminoides* was highly significantly affected by genotype, environment, and the GEI (*p* < 0.001). The sum of squares generated by the content changes based on genotype, environment, and GEI was 191.09, 78.48, and 296.98, respectively. The results showed that different genotypes of *G. jasminoides* had different requirements and adaptability to the environment, and the GEI was the main factor causing the variation in content, followed by genotype.

**TABLE 3:** Analysis of genotype–environment interactions (GEIs)

Source of variation	df	SS	MS	*F*	*p* *
Genotype	10	191.090	19.109	14.2068	<.001
Environment	2	78.482	39.241	54.4560	<.001
GEI	20	296.975	14.849	11.0395	<.001
PC1	11	209.631	19.057	14.17	<.001
PC2	9	87.34379	9.705	7.22	<.001
Residual	60	80.703	1.345		

Abbreviations: df, degrees of freedom; MS, mean squared; SS, sum of squares.

* *p* < 0.01 indicates extreme significance.

The stability of genotypes can be determined by PCA scores and AMMI stability values (ASVs) (Table 4). The closer the PC2 score was to zero, the more stable the genotype was in the test environment. Under PCA, the stability of each genotype was G4 > G7 > G5 > G1 > G6 > G3 > G11 > G8 > G2 > G10 > G9. During the ASV analysis of AMMI, the genotype with the lowest ASV score was considered the most stable. The stability of each genotype was G7 > G8 > G4 > G10 > G1 > G3 > G6 > G2 > G5 > G9 > G11. Combining the two evaluation results, HC20 (G4) and YF4 (G7) had the strongest stability.

**TABLE 4:** Analysis of additive main effects and multiplicative interactions (AMMI) principal component axis scores and stability values

Code	PC1	PC2	ASV	rASV
G1	−0.61	0.44	1.525	5
G2	0.74	−0.79	1.953	8
G3	−0.68	0.67	1.763	6
G4	0.48	−0.31	1.204	3
G5	1.34	0.43	3.256	9
G6	0.77	0.62	1.943	7
G7	0.13	−0.34	0.450	1
G8	0.26	0.72	0.942	2
G9	−1.35	−1.25	3.481	10
G10	0.42	−0.89	1.345	4
G11	−1.50	0.70	3.669	11

Abbreviations: ASV, AMMI stability value; PC1, Interaction principal component 1; PC2, Interaction principal component 2.

**FIGURE 1:**
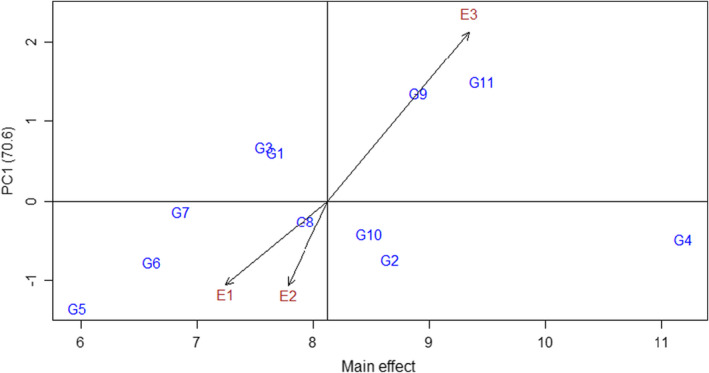
Additive main effects and multiplicative interactions (AMMI) model biplot

The average content of crocin was taken as the X‐axis, and interaction principal component 1 (IPCA1) was decomposed by G × E as the Y‐axis to make the biplot of the AMMI model (Figure 1). In the horizontal direction, the larger the abscissa is, the higher the genotype content; in the vertical direction, the closer the IPCA1 value is to 0, the more stable the genotype content. The crocin contents in the different genotypes followed the order G4 > G11 > G9 > G2 > G10 > G8 > G1 > G3 > G7 > G6 > G5. The more stable genotypes were YF4 (G7), YF5 (G8), YF7 (G10), and HC20 (G4). Based on the results of content and stability studies, YF4 (G7) was the most stable, but its crocin content was low. GY8(G11) had the highest crocin content, but its stability was poor. Therefore, of all the gardenia genotypes in this study, HC20 (G4) was both high‐yielding and stable. Therefore, HC20 (G4) should be considered a high‐yielding and stable genotype among the *Gardenia jasminoides* genotypes in this study.

### 3.3 GGE biplot analysis

#### 3.3.1 Adaptability analysis based on GGE biplot

**FIGURE 2:**
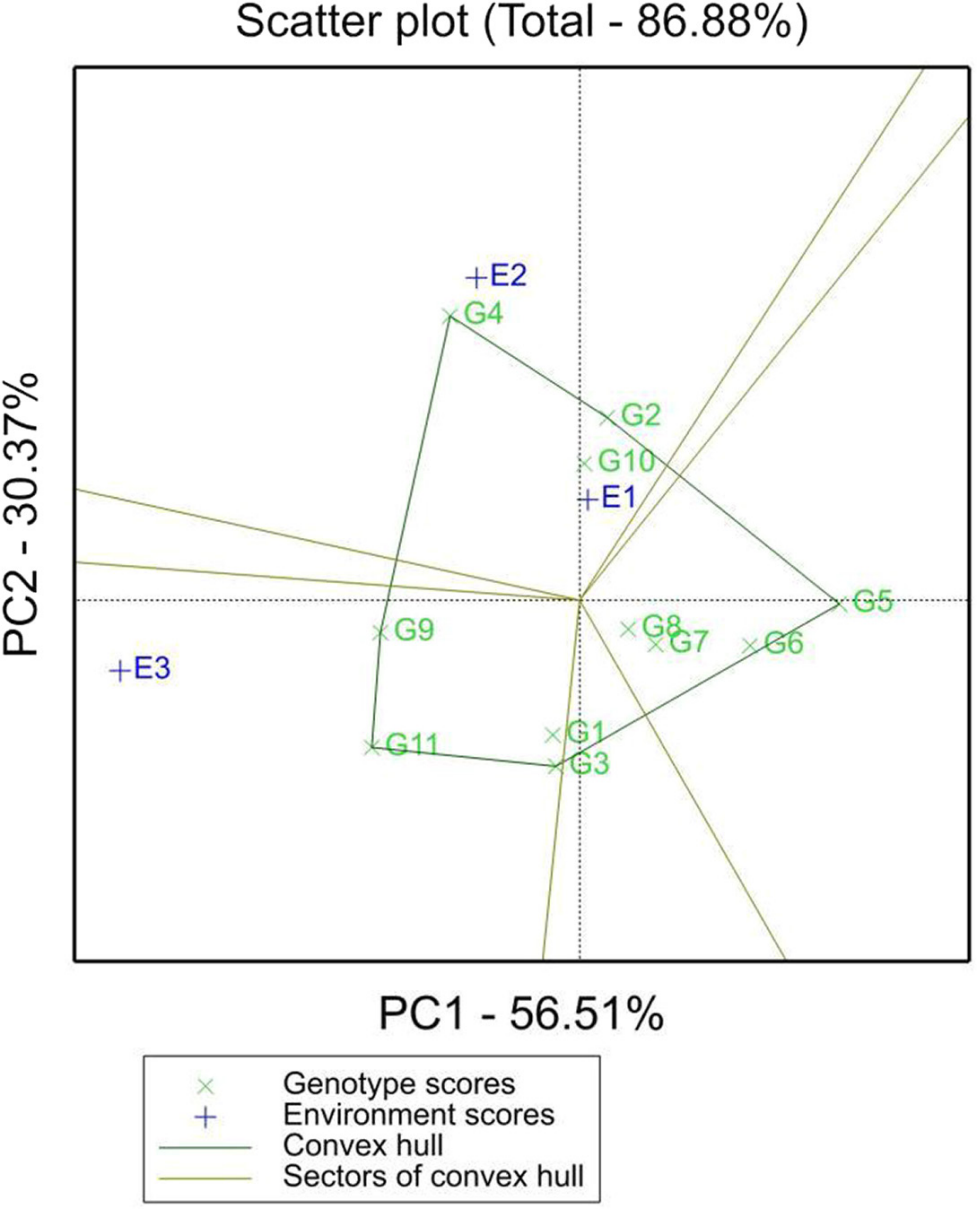
Adaptability of the crocin contents in different gardenia genotypes was analyzed based on the genotype × environment interaction (GGE) biplot

The GGE was used to construct a model to analyze the adaptability of the GEI. The varieties with the highest yield in each location and the ecological zone division of the location are explained according to the GEI. The points farthest from each direction were connected into a polygon by straight lines, and the biplot was divided into six sectors. Varieties were mainly distributed in three sectors, and the environment was divided into two sectors, with Zhangshu (E1) and Fengcheng (E2) as one sector and Gongqingcheng (E3) as the other sector (Figure 2). The variety located at the top corner of the polygon was the best genotype in each environment in the sector and was suitable for planting. Comprehensive analysis showed that the HC20 (G4) genotype had the highest crocin content in the Zhangshu (E1) and Fengcheng (E2) environments, while the GY8 (G11) genotype had the highest crocin content in another environment.

#### 3.3.2 Distinguishing force and representative analysis of test points

**FIGURE 3:**
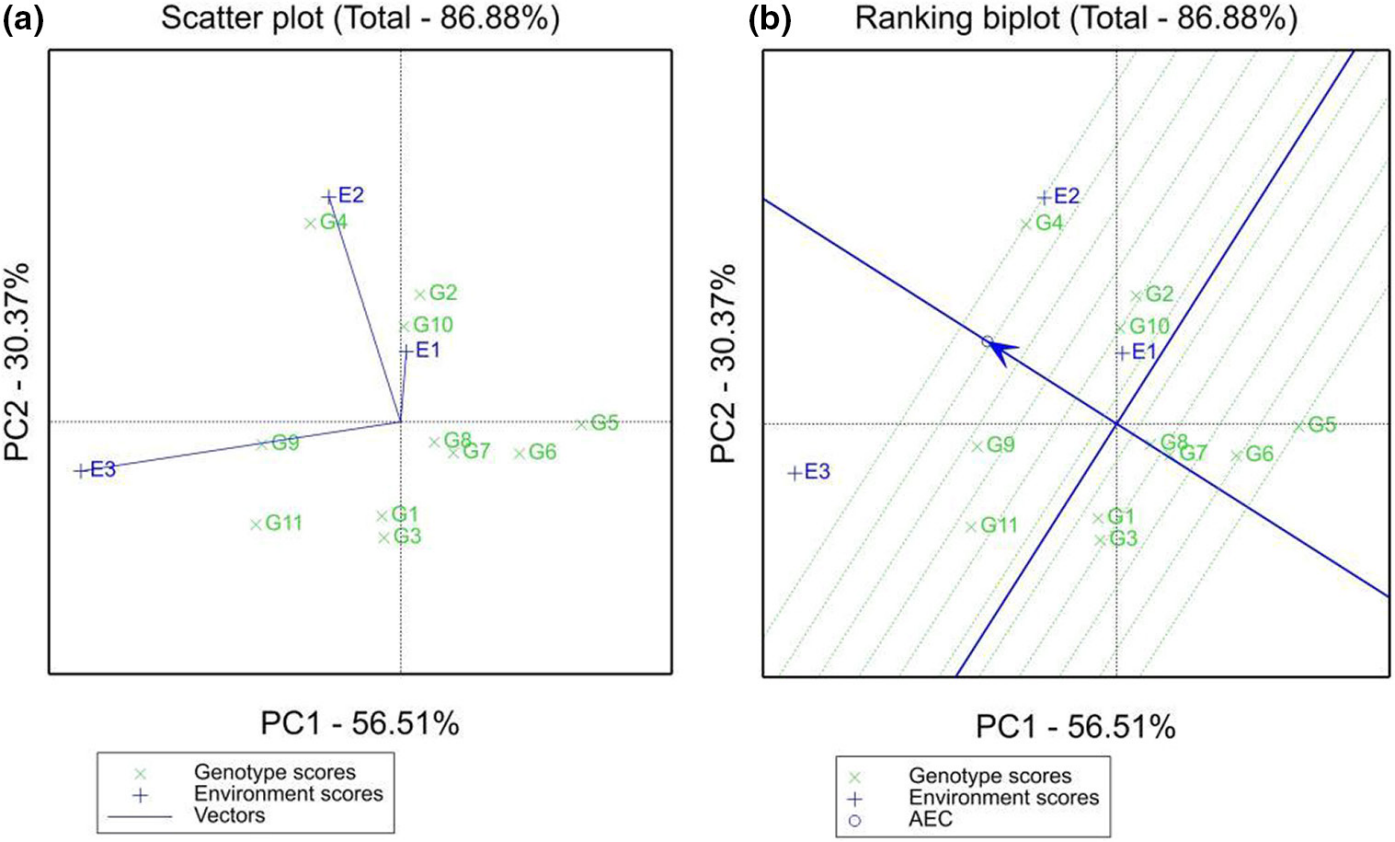
(a) Distinguishing force analysis of test points based on the genotype × environment interaction (GGE) biplot analysis; (b) Representative analysis of test points based on the GGE biplot analysis

Figure 3a shows the ability of the test site to distinguish varieties, and the longer the line segment from the center to each environment is, the stronger the differentiation ability. The results showed that Gongqingcheng (E3) and Fengcheng (E2) were far superior to Zhangshu (E1). Figure 3b shows the representativeness of each environment. The angle of the line segment of the test point and the average environment axis represent the measurement of the target environment. A smaller line segment angle between the environmental test point and the mean environmental axis indicates more representativeness. The results showed that the environmental representativeness followed the order Fengcheng (E2) > Gongqingcheng (E3) > Zhangshu (E1). Therefore, comprehensive discrimination and representative analysis indicated that the Fengcheng and Gongqingcheng test sites can be used as optimal genotype selection test sites.

#### 3.3.3 Optimal genotype screening based on GGE biplot

**FIGURE 4:**
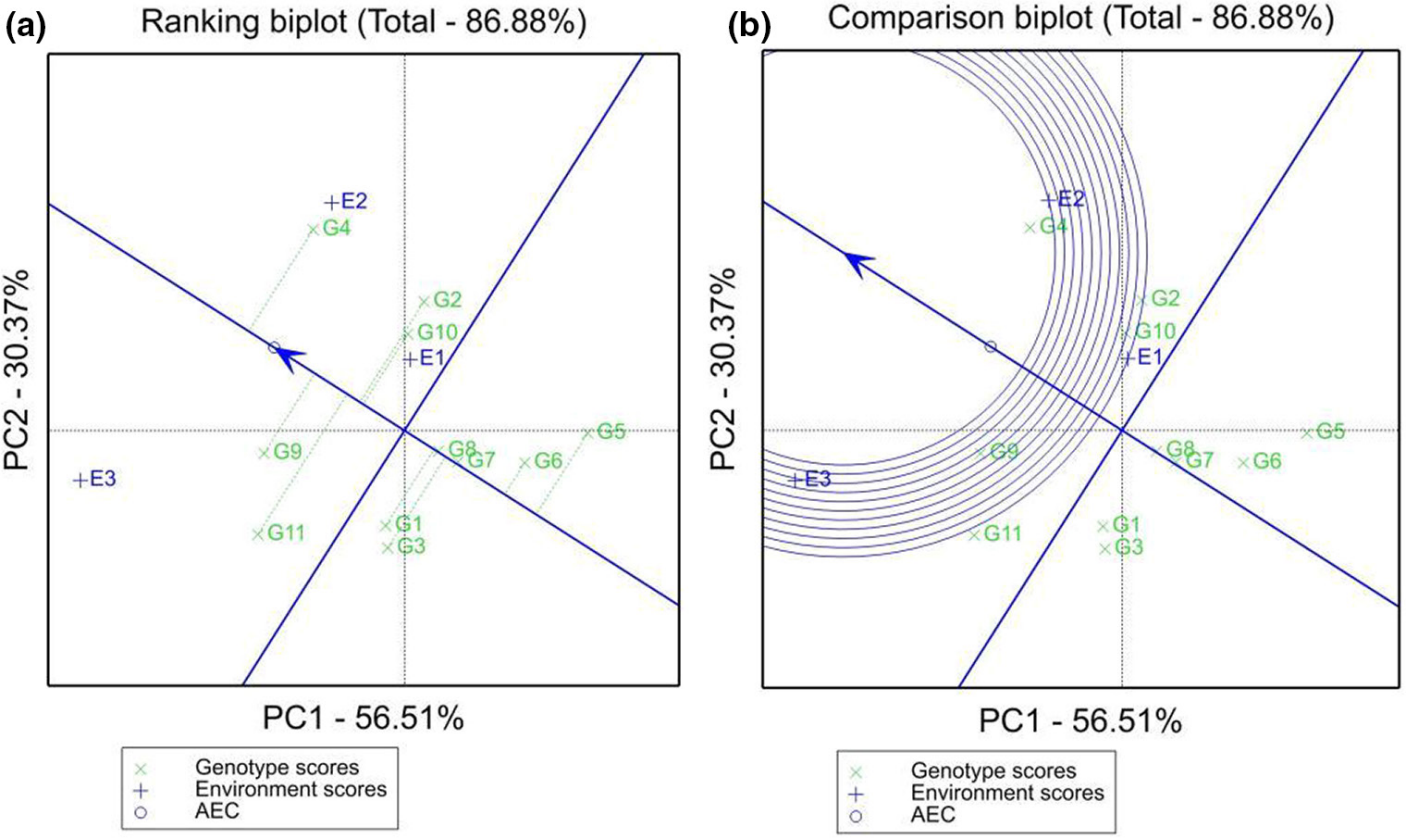
(a) Analysis of high yield and stable genotypes based on the genotype × environment interaction (GGE) biplot; (b) Analysis of optimal genotypes based on the GGE biplot. The figure should be used for the graphical table of contents and considered for the cover of the publication.

The straight line with an arrow in Figure 4a is the average environment axis (AEA). The closer the genotype is to the positive direction, the higher the yield, and a shorter genotype‐to‐environment axis perpendicular line indicates more stable yield. Among all genotypes, the highest crocin content was found for HC20 (G4), and only this genotype exceeded the average value. The lowest concentrations were found for YF2 (G5) and YF3 (G6). Among all genotypes, YF4 (G7) and YF5 (G8) had the strongest stability, while GY8 (G11) had the lowest stability.

Based on the stable and high‐yielding genotypes of *G. jasminoides*, an optimal variety screening map (Figure 4b) was constructed. The smaller the circle for each genotype is, the better the comprehensive fertility and stability of the genotype. Therefore, the variety with the best comprehensive performance was HC20 (G4), which was an ideal genotype among the tested genotypes.

## 4 DISCUSSION

Crocin synthesis and accumulation are determined by a combination of genetic and environmental factors. AMMI analysis of variance (ANOVA) showed that the GEI was the main factor affecting the overall variation in crocin content and yield of *G. jasminoides* fruit, followed by genotype, with the environmental impact being the least. It is speculated that the crocin content in *G. jasminoides* can be increased by genetic improvement under suitable environmental conditions. Previous studies showed that the GEI and genotype had the greatest influence on flavonoid and triterpene contents in leaves of *Cyclocarya paliurus* (Zhou et al., 2021). Shamloo et al. (2017) showed that the GEI effects on the secondary metabolite stigmasterol and total flavonoid contents in wheat played a major role in varietal differences, which was consistent with the results of this study. However, inconsistent with the results of this study, Lv et al. (2013) found that the carotenoids in wheat flour were mainly affected by the environment, and Röhlig et al. (2009) also showed that the environment had a more significant impact than the genetic background on corn grain metabolites. This could be due to differences in species and chemical composition or genetic types and environments becoming more diverse.

The AMMI model is widely used in stability analysis. At present, the definition of genotype stability varies. Becker and Leon (1988) believed that the genotype with the smallest variation in different environments was the most stable. However, Yan et al. (2000) pointed out that the second principal component (IPCA2) represents varietal stability. At the same time, the ASV method has been widely used in the assessment of crop yield stability (Das et al., 2010; Farshadfar, 2008). However, after combining several evaluation methods, HC20 (G4) and YF4 (G7) were found to be relatively stable genotypes. Based on the biplot of the AMMI model, HC20 was the genotype with both high and stable crocin production.

In most cases, GEI complicates the breeding, testing, and selection of superior genotypes. Significant GEI effects will cause large differences in genotypes in different environments under changes in climate and soil factors, and GGE biplots are very important for assessing adaptability and the stability of performance (Hagos & Abay, 2013). Therefore, when the impact of GEI is strong, breeders usually use the GGE biplot model to select cultivars that adapt to each environment or varieties with a wide range of adaptations (Acharya et al., 2010).

The GGE biplots can comprehensively analyze genotype effects, aid in the division and evaluation of ecological environments, and better identify representative environments and genotypes (Mitroviã et al., 2012). It is expected that, in plant breeding, stable clones will be selected in different environments (Lal et al., 2017). In this study, the HC20 (G4) genotype was the clone with the best comprehensive yield and stability and could be used as the optimal genotype for promotion and cultivation in different environments. The results are consistent with those of the AMMI model. GY8 (G11) was the genotype with the second highest crocin content, but its stability was poor. The content of GY8 (G11) in Gongqingcheng (E3) was approximately twice those in Fengcheng (E2) and Zhangshu (E1). The high yield and stability of key secondary metabolites are important goals pursued by Chinese medicinal plant breeders, but it is difficult to obtain perfect combinations in production practice. Only stable varieties with high yields can be widely planted, while low‐yielding varieties cannot be widely planted even if they have stable yields (Zhang et al., 2015). Some varieties with a wide range of environmental stabilities are poor yielding, but in some specific environments, they produce outstanding results, have high adaptability, and are suitable for local promotion. Therefore, the *G. jasminoides* line GY8 can be widely planted in the Gongqingcheng area.

The optimal environment is defined as a group of locations or environments that continuously result in the same optimal genotype or varietal characteristics. The investigation of the optimal environment is an important issue in multienvironment research and is a prerequisite for meaningful variety evaluation and recommendation (Yan et al., 2000). In this study, the Fengcheng and Gongqingcheng test sites could be used as the optimal test sites for genotype selection based on the comprehensive analysis of distinguishing forces and representativeness. Many studies have shown that the synthesis and accumulation of plant secondary metabolites are easily affected by environmental factors (Liu et al., 2015; Zhang et al., 2018). According to relevant studies, annual sunshine duration, annual precipitation, and relative humidity are important environmental factors affecting the synthesis and accumulation of crocin in *G. jasminoides* (Deng et al., 2016; Liu et al., 2022). Therefore, the interaction mechanism between environment and plant is complex and diverse. In this study, only three test sites were established, and the content of crocin in 11 different genotypes of *G. jasminoides* was comprehensively evaluated. However, the interaction mechanism between crocin synthesis and environment in *G. jasminoides* was not thoroughly studied, so it is urgent for us to further design the experimental study.

## 5 CONCLUSION

In conclusion, the content of the secondary metabolite crocin in *G. jasminoides* fruit was highly affected by the GEI. Based on distinguishing forces and representativeness analysis, the Fengcheng and Gongqingcheng test sites could be used as the optimal genotype selection test sites. In terms of genotype evaluation, the results of the AMMI biplot method and GGE biplot method were basically consistent. It was suggested that the HC20 genotype of *Gardenia jasminoides* could be popularized and cultivated as a dominant genotype in the test area, and the GY8 genotype could be widely planted in the Gongqingcheng area to achieve high‐quality and efficient crocin output.

## FUNDING INFORMATION

This research was funded by the National Natural Science Foundation of China, grant number 31760220.

## CONFLICT OF INTEREST

The authors declare that there are no conflicts of interest associated with this paper.
